# Genetic Analysis of 430 Chinese *Cynodon* *dactylon* Accessions Using Sequence-Related Amplified Polymorphism Markers

**DOI:** 10.3390/ijms151019134

**Published:** 2014-10-21

**Authors:** Chunqiong Huang, Guodao Liu, Changjun Bai, Wenqiang Wang

**Affiliations:** Tropical Crops Genetic Resources Institute, Chinese Academy of Tropical Agricultural Sciences, Key Laboratory of Crop Gene Resources and Germplasm Enhancement in Southern China, Ministry of Agriculture, Danzhou 571737, China; E-Mails: baichangjun@126.com (C.B.); wwqnmg@163.com (W.W.)

**Keywords:** *Cynodon dactylon*, genetic variation, SRAP markers

## Abstract

Although *Cynodon dactylon* (*C. dactylon*) is widely distributed in China, information on its genetic diversity within the germplasm pool is limited. The objective of this study was to reveal the genetic variation and relationships of 430 *C. dactylon* accessions collected from 22 Chinese provinces using sequence-related amplified polymorphism (SRAP) markers. Fifteen primer pairs were used to amplify specific *C. dactylon* genomic sequences. A total of 481 SRAP fragments were generated, with fragment sizes ranging from 260–1800 base pairs (bp). Genetic similarity coefficients (GSC) among the 430 accessions averaged 0.72 and ranged from 0.53–0.96. Cluster analysis conducted by two methods, namely the unweighted pair-group method with arithmetic averages (UPGMA) and principle coordinate analysis (PCoA), separated the accessions into eight distinct groups. Our findings verify that Chinese *C. dactylon* germplasms have rich genetic diversity, which is an excellent basis for *C. dactylon* breeding for new cultivars.

## 1. Introduction

The genus *Cynodon* Richard, which comprises nine species and 10 varieties, originated from tropical and subtropical areas [1]. Three species of the *Cynodon* taxa—namely *Cynodon arcuatus* (*C. arcuatus*) J.S. Presl ex C.B. Presl, *Cynodon dactylon* (*C. dactylon*, L.) Pers. var. dactylon and *C. dactylon* var. biflorus Merino—are listed in Chinese Floral acta. *C. dactylon* (common bermudagrass), the most widespread species in the genus is widely adapted and geographically distributed throughout the world between latitudes of 45°N and 45°S [1,2], and extends up to approximately 53° N latitude in Europe [3]. It is used extensively as pasture grass, turfgrass and for soil conservation throughout this region, and makes up the major turfs of public parks, golf courses, and sports fields in many countries. As high-quality turf, *C. dactylon* has recuperative potential, good color, high density, high tolerance to drought and salinity, and wear-tolerance [4]. There is extensive *C. dactylon* germplasm in China. *C. dactylon* is widely distributed from tropical Hainan Island, the most southern region of China to Beijing (in northern China). In the far northwestern part of China, *C. dactylon* is sparsely distributed in the southern and northern oasis plains in Xinjiang province [5]. In southwest China, it appears at approximately 3000 m elevation in Yunnan province, and in some valleys of Tibet. The wide geographic distribution suggests a potential for large variation in Chinese native *C. dactylon* germplasm. Although the widespread occurrence of *Cynodon* in China is well documented, there is little information on the kinds and extent “of genetic variation within the indigenous Chinese *Cynodon* germplasm pool in previous studies. The diversity within this extensive germplasm is just beginning to be evaluated and exploited for the improvement of turfgrass in China. As exploitation and evaluation of germplasms for continuous improvement of turfgrass continues, *C. dactylon* has become the focus of several breeding programs throughout the world. Recently, the genetic relationship between wild *C. dactylon* accessions collected from China and elsewhere around the world has been determined [6]. Results indicated that the Chinese accessions constitute a genetically distinct germplasm source when compared to European, African and Australian accessions, indicating an unexploited source of genetic diversity for *C. dactylon* improvement.

Turfgrass diversity studies have traditionally focused on morphological features such as density, leaf texture, leaf color, leaf and stolon length and width, and internode length and width [7]. In recent years, molecular techniques have been developed to complement traditional morphological methods in evaluating genetic diversity [8]. Different DNA markers have been used in studies of genetic diversity, relatedness, phylogeny, and in identifying off-types of *Cynodon* cultivars*.* DNA amplification fingerprinting (DAF) [9] has been used to study relatedness of some genotypes (clonal plants) of *C. transvaalensis*, *C. dactylon*, and hybrid derivatives of the two species [10,11], detect off-types originating from sod contamination instead of somatic mutation, and to identify the cultivars Tifway, Tifgreen, and Tifdwarf [12], Furthermore, DAF has been used to examine the relatedness of 18 *Cynodon* cultivars found in Australia [13], to assess genetic relatedness among 62 *Cynodon* accessions representing eight species [14], to assess the genetic relatedness of 17 seed-propagated turf-bermudagrass cultivars [15], and to determine the degree of genetic relatedness of Chinese cultivars with the existing vegetative cultivars commonly grown in the United States [16]. Randomly amplified polymorphic DNA (RAPD) [17] was used to evaluate diversity of 75 *C. dactylon* accessions collected from different regions of Iran [18]. Expressed sequence tag–simple sequence repeats (EST–SSR) [19] have also been used to characterize genetic diversity of 690 *Cynodon* accessions [20]. Amplified fragment length polymorphism (AFLP) [21] has been used to differentiate *C. dactylon* genotypes and explore their relationships [22], to detect the genetic diversity among forage bermudagrass cultivars [23], and to assess molecular genetic variation and genetic relatedness among 28 *C. dactylon* var. *dactylon* accessions originating from 11 countries on four continents (Africa, Asia, Australia and Europe) [6]. In addition, AFLP has been used to assess variations among 119 *Cynodon* accessions collected from 11 Chinese provinces [24], and to measure the genetic relatedness among entries of the *Cynodon* clonal forage bermudagrass core collection and seven commercial forage cultivars [25].

Of these methods, RAPD is one of the simplest, but has poor reproducibility. Though the AFLP method has good reproducibility and high polymorphism, its operation is very elaborate and the costs are relatively high. The method based on analysis of EST and SSR requires prior knowledge of the genome sequences of the organism to design specific primers. In comparison, sequence-related amplified polymorphism (SRAP) markers [26] overcome most of these limitations. SRAP is aimed at preferentially amplifying open reading frames (ORFs), which are coding sequences in the genome. It can disclose numerous co-dominant markers with a large amount of polymorphic loci and allows easy isolation of bands for sequencing. This technique can generate more polymorphic fragments for the assessment of genetic diversity than the SSR, inter-simple sequence repeat (ISSR) and RAPD markers [27]. Ferriol and co-authors showed that the information generated by SRAP markers was more consistent with the morphological variability and evolutionary history of the morphotypes than that obtained by using AFLP markers [28]. Due to advantages such as production of highly specific polymorphic fragments, easy manipulation, reliability, moderate throughput, and ease in sequencing selected bands, this approach has been successfully used for a wide range of analysis in various species, including *Brassica oleracea*, *Buchloe dactyloides* (Nutt.) Englem, *Cucurbita pepo*, *Pennisetum purpureum* Schumach, *Lactuca* sp*.*, and *Salvia miltiorrhiza* Bge [26,27,28,29,30,31].

Although previous research has provided preliminary data regarding genetic diversity at the species level, studies that conduct the systematic analysis of the genetic diversity and population structure of Chinese *C. dactylon* accessions are limited. Considering the advantages of SRAP markers, we used this method to assess the genetic diversity of 430 *C. dactylon* accessions collected from 22 provinces in China*.*

## 2. Results and Discussion

### 2.1. Sequence-Related Amplified Polymorphism (SRAP)

Of the 100 SRAP primer pairs evaluated for their ability to amplify *C. dactylon* DNA, 85 primer pairs were rejected because they either produced no amplification or displayed monomorphic patterns. Fifteen primer pairs from the original 100 primer pairs were selected based on the polymorphic and reproducible bands they produced.

The 15 primer pairs amplified 481 reproducible fragments, ranging in size from 260–1800 base pairs (bp), of which 481 (100%) were polymorphic. The highest number of amplification products was obtained using the primer pairs Me9–Em1 (Me9 is the forward prime name, Em1 is the reverse prime name) and Me11–Em8, and the lowest with Me10–Em6 (Me11 and Me10 are the forward prime names, Em8 and Em6 is the reverse prime names). The average number of bands among the 15 primer pairs was 32. The number of polymorphic fragments for each primer pair varied from 25–38, with an average value of 32.

The effective allele number (ne) ranged from 1.2283–1.5704 with an average value of 1.3952, Nei’s gene diversity (h) varied between 0.1631 and 0.3326 with an average value of 0.2461, Shannon’s index (I) varied between 0.2808 and 0.4980 with an average value of 0.3865 (Table 1).

**Table 1:** Polymorphisms detected by 15 sequence-related amplified polymorphism (SRAP) primer pairs in 430 accessions of *Cynodon dactylon* (*C. dactylon*).

Primer Pairs	Band Size (bp)	Total Bands	Polymorphic Bands	Effective Allele Number (ne)	Nei’s Gene Diversity (h)	Shannon’s Index (I)
Me1-Em1	280–750	28	28	1.4792	0.2836	0.4325
Me1-Em3	380–1600	34	34	1.3426	0.222	0.354
Me1-Em6	320–1450	33	33	1.2283	0.1631	0.2808
Me1-Em8	320–1500	28	28	1.3673	0.2297	0.3635
Me5-Em1	320–1700	37	37	1.4274	0.2636	0.4134
Me5-Em5	320–1500	30	30	1.3068	0.1864	0.2953
Me7-Em7	340–1700	28	28	1.3498	0.2261	0.3599
Me8-Em8	260–1500	27	27	1.4000	0.2541	0.402
Me9-Em1	290–1800	38	38	1.4076	0.2526	0.3965
Me9-Em5	300–1600	37	37	1.2956	0.1911	0.3139
Me10-Em1	260–1750	36	36	1.5024	0.3097	0.4757
Me10-Em4	260–1750	35	35	1.378	0.2353	0.3718
Me10-Em6	260–1600	25	25	1.3885	0.2482	0.3929
Me11-Em5	300–1500	27	27	1.4838	0.2936	0.4477
Me11-Em8	320–1700	38	38	1.5704	0.3326	0.4980
Total	–	481	481	–	–	–
Average	–	32	32	1.3952	0.2461	0.3865

### 2.2. Genetic Diversity Analysis

The genetic similarity coefficients (GSC) of the 430 accessions varied between 0.53 and 0.96 with an average value of 0.72. Greater genetic distance indicated farther genotype relatedness between the genotypes. The lowest GSC (0.53) was between A175 from Hainan province and A068 from Jiangsu province (A175 and A068 are the accessions names), which suggests that these were the least related accessions, whereas the highest GSC was 0.96, detected between accessions A167 and A166 from Shandong province (A167 and A166 are the accessions names), indicating a very close relationship. The range of GSC varied from 0.53–0.96 on seasides, 0.58–0.94 on roadsides, 0.60–0.89 in open field, and 0.67–0.93 in grassland, indicating that the largest variations exist in seaside areas and the smallest variations occur in grasslands. The GSC varied from 0.79–0.91 in north China (Beijing, Tianjin, and Hebei provinces), 0.79–0.90 in northwest China (Gansu and Shaanxi provinces), 0.77–0.94 in central China (Henan, Hubei, Hunan and Jiangxi provinces), 0.60–0.96 in eastern China (Shandong, Shanghai, Zhejiang, Anhui and Jiangsu Province), 0.70–0.93 in southwest China (Sichuan, Guizhou, Yunnan and Chongqing Province), 0.58–0.95 in south China (Guangdong, Guangxi, Fujian and Hainan Province), indicating that the largest variations exist in southwest China, followed by eastern China and southwest China, and the smallest variations occur in north China and northwest China.

**Figure 1:**
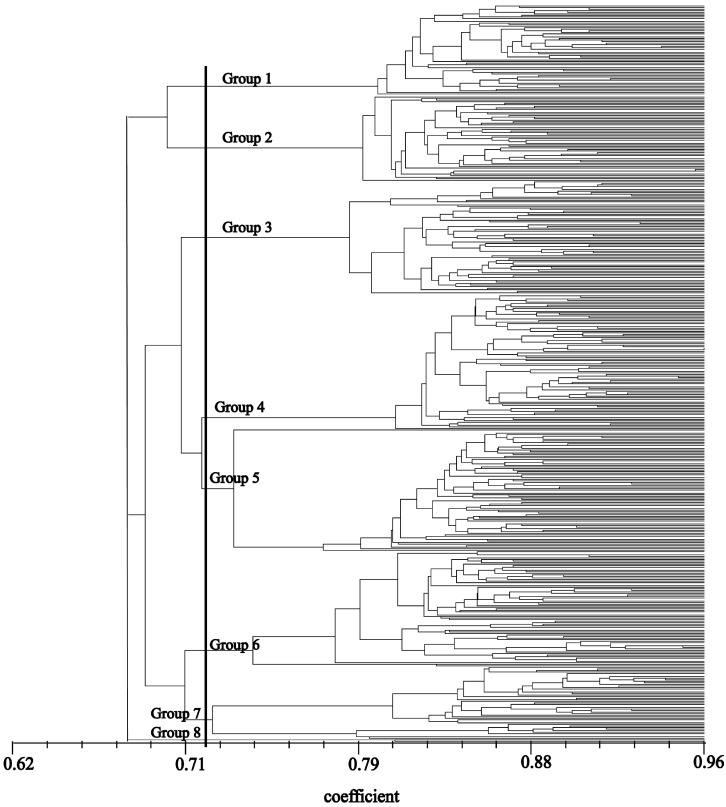
The dendrogram of 430 Chinese *C. dactylon* accessions derived from cluster analysis (unweighted pair-group method with arithmetic averages (UPGMA)) based on genetic similarity estimates from SRAP marker analysis.

A dendrogram was constructed to group the 430 accessions into eight major groups at the 0.72 similarity level (Figure 1), with most accessions from the same geographic locations or nearby regions tending to have high genetic similarity and clustering into the same groups or neighboring groups. Group 1 included 53 accessions collected from north China (Beijing, Tianjin and Hebei provinces), northwest China (Gansu and Shaanxi provinces), and central China (Henan, Hubei, Hunan and Jiangxi provinces), whereas Group 2 included 50 accessions collected from Jiangsu province of eastern China. Group 3 comprised 66 accessions from eastern China (Shanghai, Anhui and Zhejiang provinces). Group 4 consisted of 79 accessions from Shandong province of eastern China. Group 5 consisted of 71 accessions from Southwest China (Yunnan, Guizhou, Sichuan and Chongqing provinces), and was further divided into two subgroups. One subgroup contained only one accession collected from a very dry area (hillside) of Yunnan province, and was distinct from the other accessions—it had short, broad leaves, with light leaf and stolon colour, and fine stolon internode diameter. Group 6 included 67 accessions from South China (Guangdong, Guangxi and Fujian provinces), Group 7 contained 42 accessions from Hainan province, the most southern region of China. Group 8 included two accessions from Hainan province, with a GSC of 0.81, indicating close relationships within this group.

**Figure 2:**
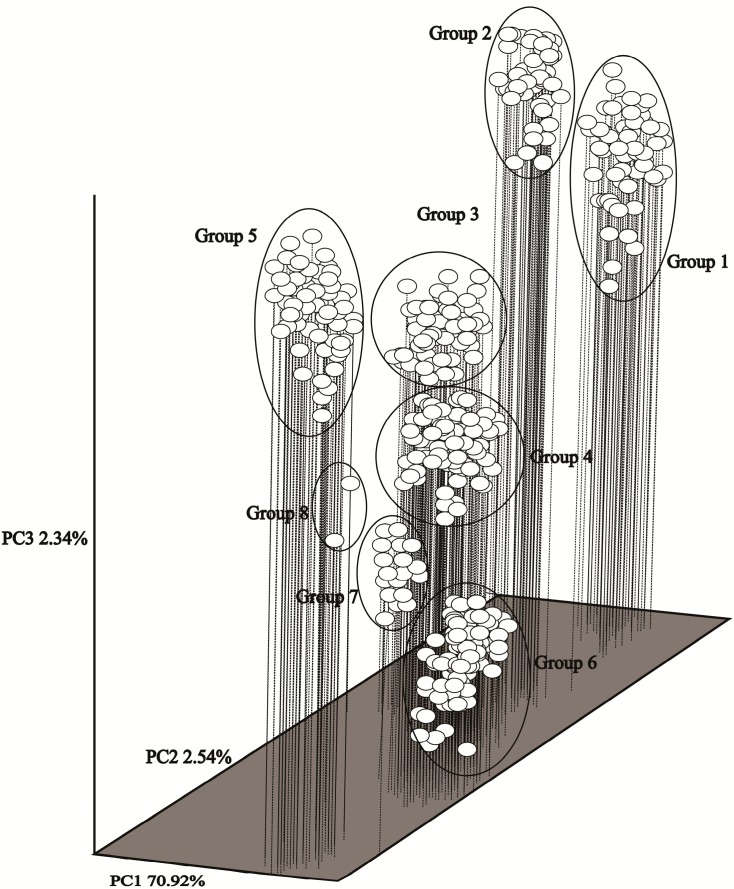
Principle coordinate analysis of 430 Chinese *C. dactylon* accessions based on the genetic similarity matrix derived from the combined data of SRAP markers.

Principle coordinate analysis (PCoA) was conducted based on the genetic resemblance matrix to further understand the ecological distribution of different accessions. Figure 2 presents the distribution of the different accessions according to the three principal axes of variation using PCoA. The percentages of variance revealed by principle component 1 (PC1), principle component 2 (PC2) and principle component 3 (PC3) were 70.92%, 2.54% and 2.34%, respectively, which were consistent with the results of unweighted pair-group method with arithmetic averages (UPGMA) cluster analyses.

### 2.3. Discussion

To the best of our knowledge, this is the first report where Chinese *C. dactylon* accessions collected from a very wide range of natural habitats were evaluated by using the SRAP molecular marker technique. Previous studies examined only 119 Chinese accessions collected from 11 provinces in China [6], whereas our study included 430 Chinese accessions collected from 22 provinces. Although our study examined a wide range of accessions from China, it suffers from the limitation that no accessions were collected from Xinjiang province, which is the major geographic region of *C. dactylon*, and has a distinct environmental condition. Several studies have suggested that all the germplasms of the species growing in major geographic regions should be collected to fully understand genetic variation [6,32,33]. Taking this into account, we will continue collecting accessions from Xinjiang province and other major geographic regions where *C. dactylon* occurs. Wu and co-authors, Huang and co-authors and Yerramsetty and co-authors support the position that the Chinese *C. dactylon* accessions represent a distinct and valuable source of germplasm for variety development, which should be used to further increase the genetic diversity of *C. dactylon* in China [6,16].

In the present study, we found a high level of polymorphism (100%) among various accessions, which is consistent with the findings of previous studies [33,34], and further confirms that the SRAP marker technique generates highly reproducible DNA profiles for *C. dactylon* accessions. The high levels of polymorphism may partially be attributed to the following: (1) the *C. dactylon* accessions have their own unique natural habitats since they were collected from a very wide geographic range; (2) the SRAP marker technique is designed to amplify ORFs. We also observed that the SRAP method produced more polymorphic bands per primer pair (32 bands per primer) compared to other methods described in previous reports [35]. This difference may be due to the types of specimen analyzed and the different detection methods employed. Furthermore, we found that the number of total bands and polymorphic bands produced by each primer varied widely. There seemed to be no correlation between the number of bands amplified and the degree of polymorphism. A similar phenomenon was also observed when using ISSR analysis in a previous study [36].

The 430 *C. dactylon* accessions were clustered into eight groups by UPGMA on the basis of 431 polymorphic fragments. Genetic similarity coefficients (GSC) varied among the 430 accessions from 0.53–0.96. This range is consistent with those found in several other studies on the same plant. One report showed a GSC range of 0.53–0.98 [6], whereas another report showed a range of 0.50–0.98 [37]. However, these values are higher than the range of 0.42–0.94 reported by Kang and co-authors [38], but lower than the range of 0.65–0.99 reported by Wu and co-authors [24]. The GSC values obtained in our study demonstrated that the level of genetic diversity was relatively high among *C. dactylon* accessions. The genetic locations of the clusters obtained from PCoA also demonstrated wide genetic variability among the clusters. These results suggest that a high level of polymorphism exist in wild accessions.

In summary, accessions examined in the present study were collected from a wider geographic region than those in earlier studies, although limited numbers of accessions were used from some key regions, e.g., Xinjiang province. Therefore, future research studies on the genetic diversity of *C. dactylon* should involve more extensive germplasm collection. The SRAP marker technique has advantages in terms of convenience, good reproducibility, and high polymorphism and can therefore be used more extensively in genetics research. Furthermore, the molecular relationships generated from this study should be useful in breeding programs for *C. dactylon*.

## 3. Experimental Section

### 3.1. Plant Materials

**Figure 3:**
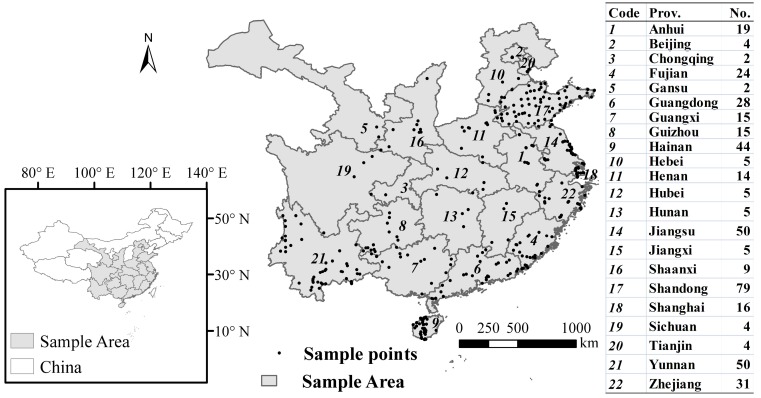
A map showing the collection sites for the Chinese *C. dactylon.*

A total of 430 wild *C.* *dactylon* accessions collected from 22 provinces in China, ranging from tropical Hainan Island to the temperate climatic region around Beijing, between 3° and 18°N latitudes and between 98°E and 122°E longitudes were used in this study (Table 2, Figure 3). All Chinese accessions were wild accessions, didn’t include any commercial bermudagrasses varieties, and were determined to be *C. dactylon* based on morphological characteristics by three plant classification experts.

**Table 2:** Geographical origins of 430 *C. dactylon* accessions investigated in the present study.

Accession Number	Origin	Percentage of Accession Number (%)
13	North China (Beijing, Tianjin and Hebei Province)	3.02
11	Northwest China (Gansu and Shaanxi Province)	2.56
29	Central China (Henan, Hubei, Hunan and Jiangxi Province)	6.74
71	Southwest China (Sichuan, Guizhou, Yunnan and Chongqing Province)	16.51
195	Eastern China (Shandong, Shanghai, Zhejiang, Anhui and Jiangsu Province)	45.35
111	South China (Guangdong, Guangxi, Fujian and Hainan Province)	25.81

Each accession was originally collected from grassland, roadside, sea side or open field, and was grown in three 20-cm diameter pots in the greenhouse under uniform conditions at the Tropical Crops Genetic Resources Institute, Chinese Academy of Tropical Agricultural Sciences (Hainan Island). The accessions were homogeneous before leaves were collected for DNA isolation.

### 3.2. DNA Extraction

Fresh and healthy leaves (100 mg) of each genotype were collected from at least two pots (vegetative clones) of greenhouse grown plants. Total genomic DNA was isolated following the hexadecyltrimethylammonium bromide (CTAB) DNA extraction procedure with some modification [39]. The quality and quantity of genomic DNA were determined visually based on band intensities following standard horizontal electrophoresis on 1.0% (*w*/*v*) agarose gels (Trans transgen biotechnology, Beijing, China). The concentration was adjusted to 20 ng/μL for PCR amplification.

### 3.3. SRAP Reactions

One hundred different primer pairs from Shenggong Inc. (Shanghai, China) were tested for PCR analysis, starting from 10 forward and 10 reverse primers (Table 3) [26]. A total of 100 SRAP primer pairs were screened on three randomly selected accessions. Primer pairs were excluded when their banding patterns were difficult to score or failed to amplify any products. Of these 100 primer pairs, 15 primer pairs were chosen for further analysis based on their good amplification capability (Table 4).

Each 25 μL PCR reaction mixture consisted of 80 ng of genomic DNA, 0.4 μM primer, 250 μM deoxy-ribonucleoside triphosphate (TaKaRa Biotechnology, Dalian, China), 1.5 mM magnesium chloride (MgCl_2_, TaKaRa Biotechnology, Dalian, China), 1.5 units of Taq polymerase (TaKaRa Biotechnology, Dalian, China), and 2.5 μL of 1× PCR buffer (TaKaRa Biotechnology, Dalian, China). The mixture was overlaid with 20–30 μL mineral oil before thermal cycling was commenced. DNA amplification was carried out on a TaKaRa PCR Thermal Cycler Dice™ (TaKaRa Biotechnology, Dalian, China) with the following conditions: initial denaturation at 94 °C for 4 min, followed by five cycles of 1 min denaturation at 94 °C, 1 min annealing at 35 °C and 30 s of elongation at 72 °C. In the following 30 cycles, 1 min denaturation at 94 °C, 1 min annealing at 50 °C, and 30 s elongation at 72 °C were performed, ending with an elongation step of 10 min at 72 °C. The amplified products were kept at 4 °C until they were loaded onto the gel. The amplification products were fractionated via 6% (*w*/*v*) polyacrylamide gel electrophoresis (PAGE, Shenggong Inc., Shanghai, China)in 1× TBE (0.09 mol/L Tris-H_3_BO_3_, 0.002 mol/L EDTA, pH 8.0, Shenggong Inc., Shanghai, China) at constant 1800 V and room temperature for 3 h and then visualized using the simplified silver staining method [40].

**Table 3:** The forward and reverse SRAP primers used in this study.

Name	Forward Primer (5'–3')	Name	Reverse Primer (5'–3')
Me1	TGAGTCCAAACCGGATA	Em1	GACTGCGTACGAATTAAT
Me2	TGAGTCCAAACCGGAGC	Em2	GACTGCGTACGAATTTGC
Me3	TGAGTCCAAACCGGAAT	Em3	GACTGCGTACGAATTGAC
Me4	TGAGTCCAAACCGGACC	Em4	GACTGCGTACGAATTTGA
Me5	TGAGTCCAAACCGGAAG	Em5	GACTGCGTACGAATTAAC
Me7	TGAGTCCAAACCGGTAG	Em6	GACTGCGTACGAATTGCA
Me8	TGAGTCCAAACCGGTAA	Em7	GACTGCGTACGAATTCGA
Me9	TGAGTCCAAACCGGTCC	Em8	GACTGCGTACGAATTCAA
Me10	TGAGTCCAAACCGGTGC	Em9	GACTGCGTACGAATTCTG
Me11	TGAGTCCAAACCGGT	Em10	GACTGCGTACGAATTAGC

**Table 4:** The 15 SRAP primer pairs used in this study.

Code	Primer Pairs	Code	Primer Pairs	Code	Primer Pairs
1	Me1-Em1	6	Me5-Em5	11	Me10-Em1
2	Me1-Em3	7	Me7-Em7	12	Me10-Em4
3	Me1-Em6	8	Me8-Em8	13	Me10-Em6
4	Me1-Em8	9	Me9-Em1	14	Me11-Em5
5	Me5-Em1	10	Me9-Em5	15	Me11-Em8

### 3.4. Data Analysis

SRAP bands throughout the gel profiles were scored visually for their presence (1) or absence (0) at least twice for each accession. Only reproducible and unambiguous SRAP fragments were used for scoring. The data were compiled in a binary data matrix using Microsoft Excel and analyzed using the numerical taxonomy and multivariate analysis system (NTSYS) program, version 2.1 (Exeter Software, Setauket, NY, USA). Simple matching coefficients were computed using the SIMQUAL module of the NTSYS program. Cluster analysis based on genetic similarity coefficients (GSCs) using the Nei and Li distance was performed according to the unweighted pair-group method with arithmetic averaging (UPGMA) in the SAHN module of the NTSYS program [38]. Principal coordinate analysis (PCoA) was performed to estimate the genetic distances among the major groups using the DCENTER and EIGEN modules of the NTSYS program. Effective allele number (ne), Nei’s gene diversity (He) and Shannon’s information index (I) were used to compute Nei’s standard genetic distance coefficients using the Popgene32 program [41].

## 4. Conclusions

This study demonstrates that the SRAP technique is a reliable tool for differentiating *C. dactylon* accessions and determining the genetic relationship among accessions. Fifteen SRAP primer pairs were used to amplify specific *C. dactylon* genomic sequences and 481 SRAP bands were generated. Genetic similarity coefficients ranged from 0.53–0.96 and cluster analysis separated the accessions into eight distinct groups based on SRAP markers.
